# Obesity-Induced Insulin Resistance Is Mediated by High Uric Acid in Obese Children and Adolescents

**DOI:** 10.3389/fendo.2021.773820

**Published:** 2021-12-03

**Authors:** Yang Niu, Qingya Tang, Xuan Zhao, Xuelin Zhao, Xiaomeng Mao, Jinye Sheng, Wei Cai, Yi Feng

**Affiliations:** ^1^ Department of Clinical Nutrition, Xinhua Hospital, School of Medicine, Shanghai Jiao Tong University, Shanghai, China; ^2^ Shanghai Key Laboratory of Pediatric Gastroenterology and Nutrition, Shanghai, China; ^3^ Shanghai Institute for Pediatric Research, Shanghai, China

**Keywords:** insulin resistance, uric acid, obesity, children, adolescents

## Abstract

**Objective:**

This study aimed to evaluate whether serum uric acid (SUA) plays a mediating role in the development of insulin resistance (IR) in obese children and adolescents.

**Methods:**

A total of 369 participants aged 4-17 years with obesity who attended the Nutrition Outpatient Clinic for Obesity at Xinhua Hospital from January 2012 to January 2019 were recruited for this retrospective study. We classified participants into two groups on the basis of HOMA-IR values: the low HOMA-IR group (< 3.16) (n = 222) and the high HOMA-IR group (≥ 3.16) (n = 147).

**Results:**

The univariate analysis found that the high HOMA-IR group had higher BMI, SUA, and fasting insulin (FINS) (*P <* 0.05). Multiple linear regression analysis and mediating effect analysis indicated that body mass index (BMI) could directly regulate FINS and HOMA-IR (both *P <* 0.05). The results from the mediating effect analysis found that UA partially played an indirect role in the link between BMI, FINS and HOMA-IR (both *P <* 0.05) but had no effect on fasting blood glucose (*P >* 0.05).

**Conclusions:**

SUA should be investigated in obesity and plays a partial mediating role in insulin resistance induced by obesity in obese children and adolescents.

## Introduction

Obesity is an increasingly serious clinical and public health problem with more than 100 million obese people worldwide (1). The prevalence of obesity is as high as 20.3% (7-12 y) and 9.6% (13-17 y), respectively, in Chinese children and adolescents (2). Obesity is a driving factor of some endocrine diseases, such as insulin resistance (IR), type 2 diabetes, hypertension, hyperuricemia, and metabolic syndrome (3–6), which places a heavy burden on patients, families, and the public health system (7). Researchers have confirmed that IR is commonly present in obese children and adolescents and is associated with an increased risk of developing type 2 diabetes in adulthood (3, 8). Therefore, identifying and modifying risk factors for IR should be beneficial in preventing diabetes and other metabolic diseases in these high-risk youth.

Obesity diagnosed by body mass index (BMI) has been reported to result in hyperuricemia in more cross-sectional studies (9, 10). Recent studies have found that hyperuricemia not only leads to gout arthritis and nephropathy but may also be related to IR, type 2 diabetes and cardiovascular morbid events (9, 11–13). Thus, SUA interacts with other factors in the modulation of obesity and IR.

However, the mechanism of IR in obese children is still not clear. In addition, to our knowledge, there is no previous epidemiologic study that investigated whether hyperuricemia exerts a mediating effect on the relationship between obesity and IR in children. Therefore, we hypothesized that a BMI-SUA-IR pathway is present in obese children and adolescents. In the current study, we examined the associations among IR, SUA and BMI with multiple linear regression analysis and mediation analysis.

## Methods

### Subjects

**Figure 1:**
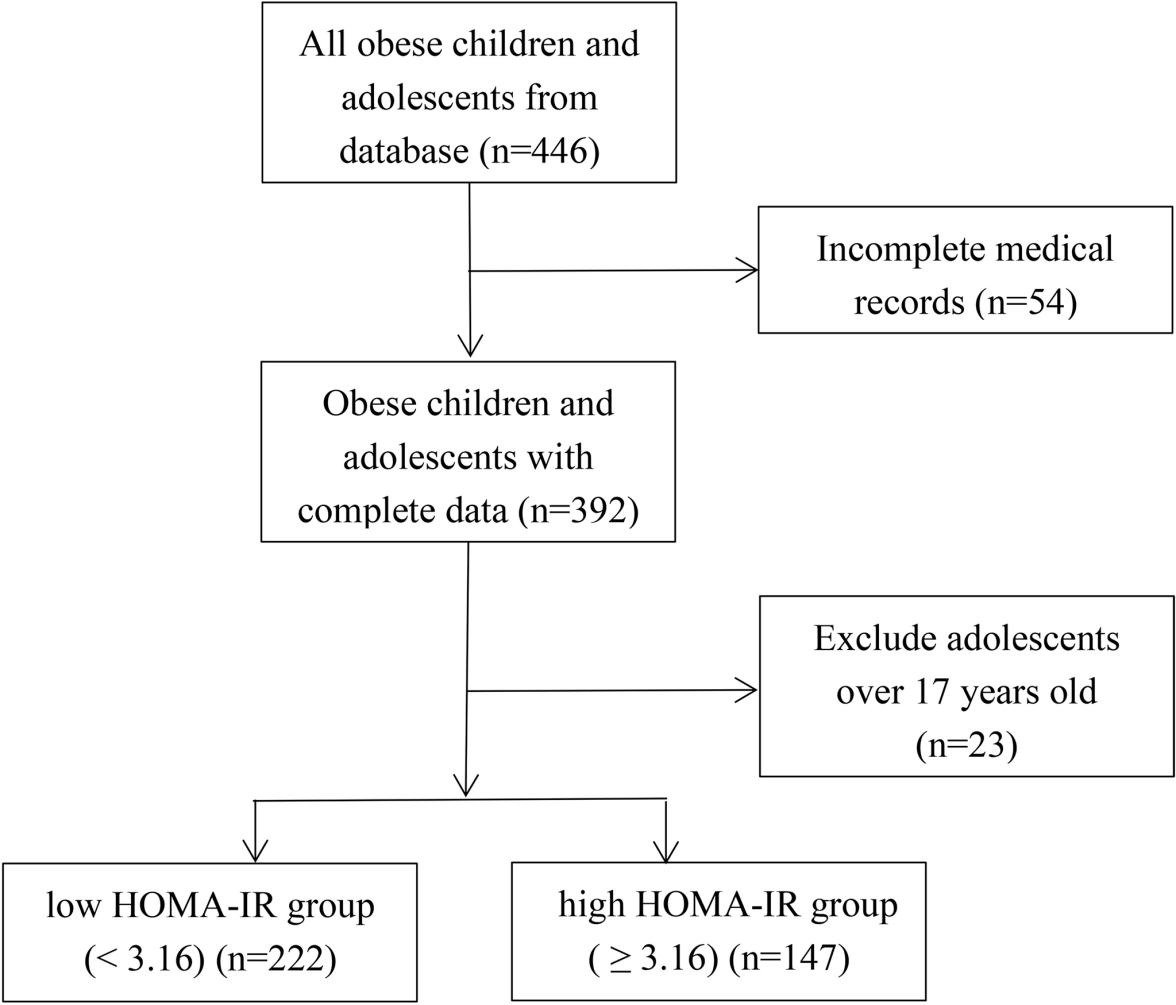
Flowchart of collecting data.

A retrospective study focused on 369 outpatients with obesity, aged 4-17 years, who attended the Nutrition Outpatient Clinic for Obesity at Xinhua Hospital from January 2012 to January 2019 (**Figure 1**). According to the World Health Organization standards, obesity is defined as BMI ≥ the 95th percentile of children of the same sex and age (14). Participants with severe kidney and liver damage and obesity caused by endocrine diseases or genetic metabolic diseases were excluded from this study. This study was reviewed and approved by the Ethics Committee of Xinhua Hospital, School of Medicine, Shanghai Jiao Tong University (No. XHEC-D-2021-113).

### Data Collection and Measurement

All data from the first clinical visit were collected at Xinhua Hospital, located in Shanghai, China. Basic characteristics, including sex, age, height, and weight, were recorded. Accordingly, BMI (kg/m^2^) was determined as weight in kg divided by height in meters squared.

Triglyceride (TG), total cholesterol (TC), high-density lipoprotein cholesterol (HDL-C), low-density lipoprotein cholesterol (LDL-C), fasting blood glucose (FBG), and fasting insulin (FINS) were tested at the first clinical visit. All of these parameters were measured in the hospital laboratory centre using a Hitachi 7180 automatic biochemical analyzer (Hitachi, Japan). Serum uric acid (SUA) was analyzed using the urase method.

Homeostasis model assessment-β (HOMA-β) and homeostasis model assessment-insulin resistance (HOMA-IR) were calculated to evaluate β-cell function and IR using the following equations: HOMA-β = 20 × FINS (mIU/L)/[FBG (mmol/L)‐3.5]%; HOMA-IR = FBG (mmol/L) × FINS (mIU/L)/22.5 (15). At present, there is no determination of the cut-off point of IR in children. Most studies have defined IR with a HOMA-IR value of 3.16 in children and adolescents (16, 17). Thus, we classified participants into two groups on the basis of HOMA-IR values: the low HOMA-IR group (< 3.16) (n = 222) and the high HOMA-IR group (≥ 3.16) (n = 147).

### Statistical Analysis

All statistical analyses were carried out using SPSS V.25.0 statistical software. Descriptive participant characteristics are presented as the mean ± SD for continuous variables and median (p25, p75) for non-normally distributed data.

For the categorical variables, the chi-square test and percentage (%) were used. Univariate analysis was performed to analyze the correlation between IR and variables and the correlation between SUA and variables. Furthermore, multiple linear regression analysis was conducted to evaluate the direct impacts of BMI and SUA on IR using different models: Model 1 (crude model), Model 2 (adjusted for age and sex), and Model 3 (adjusted for age, sex, TG, TC, HDL-C, and LDL-C) and to evaluate the direct impacts of BMI on SUA using different models: Model 1 (crude model), Model 2 (adjusted for age and sex), and Model 3 (adjusted for age, sex, TG, TC, HDL-C, LDL-C, FBG and FINS).

**Figure 2:**
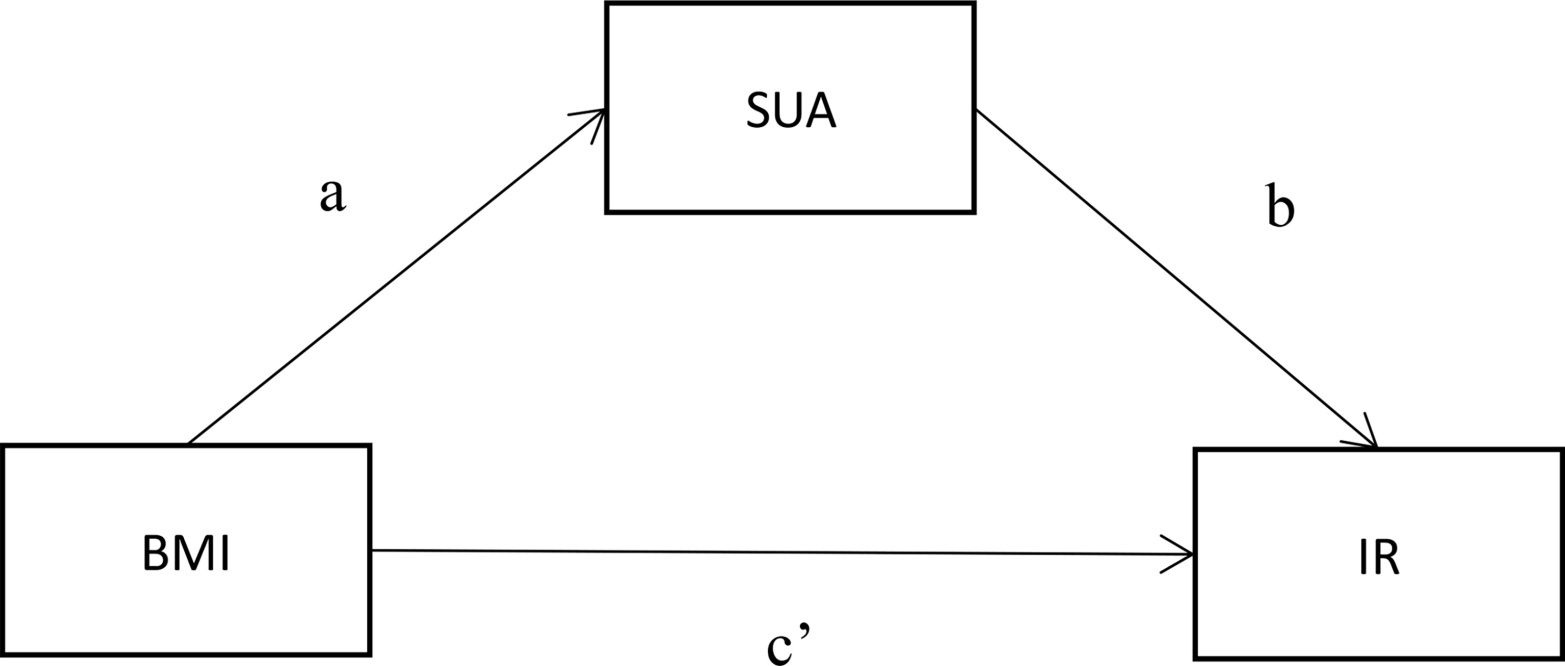
Mediation model for the association between BMI and IR with SUA as a mediator. **(A)** represents the regression coefficients for the association between BMI and SUA; **(B)** represents the regression coefficients for the association between SUA and glucose metabolism; **(C**’**)** represents the total effect between BMI and glucose metabolism.

Advanced analysis of the mediating effect was conducted by constructing two causal pathways, a BMI → SUA → IR pathway (**Figure 2**). For this pathway, BMI was fitted as an independent variable, SUA was the potential mediator, and IR was the outcome of interest. In the pathway, the “total effect” consisted of a “direct effect” (not mediated by SUA) and an “indirect effect” (completely or partly mediated by SUA). If the direct impact was significant [p(c’) < 0.05] and the indirect impact was not significant [both p(a) and p(b) > 0.05], then the link between BMI and IR was not mediated by SUA. The association between BMI and IR was fully mediated by SUA, with the indirect effects being significant [both p(a) and p(b) < 0.05] but the direct effects being not significant [p(c’) > 0.05]. When both the indirect effects and the direct effects were significant [p(a), p(b) and p(c’) < 0.05], SUA played a partial mediating effect on the relationship between BMI and IR. Path analyses were performed using Process v2.16.3 by Andrew F. Hayes (18, 19). All two-sided *P* values < 0.05 were considered statistically significant.

## Results

### Participants Characteristics and Factors Associated With IR Among the Total Sample

A total of 369 participants, with a mean age of 10.53 ± 2.43 years, were recruited in this study, including 252 (68.29%) boys and 117 (31.71%) girls. Compared with the low HOMA-IR group (< 3.16), the high HOMA-IR group (≥3.16) was older and had higher BMI, SUA, TG, TC, LDL-C, FINS, and HOMA-IR but lower HDL-C (all p < 0.05, **Table 1**).

**Table 1:** Participants characteristics in different HOMA-IR groups among total sample.

	Total	HOMA-IR		*P*
		<3.16	≥3.16	
**n**	**369**	**222**	**147**	
**Sex**				
Boy [n,(%)]	252 (68,29)	157 (70.70)	95 (64.60)	0.309
Girl[n,(%)]	117 (31.71)	65 (29.30)	52 (35.40)
**Age (y)**	10.94 ± 2.89	10.19 ± 2.89	12.09 ± 2.50	<0.001
**Height (m)**	1.52 ± 0.15	1.47 ± 0.15	1.59 ± 0.11	< 0.001
**Weight (kg)**	64.90 (50.50, 82.00)	56.98 (46.32, 71.40)	76.40 (62.40, 92.30)	<0.001
**BMI (kg/m^2^)**	28.45 ± 4.71	27.15 ± 4.16	30.42 ± 4.83	<0.001
**SUA (umol/L)**	371.00 (312.50, 454.00)	351.50 (297.50, 406.25)	415.00 (350.00, 497.00)	<0.001
**TG (mmol/L)**	1.44 ± 0.94	1.33 ± 0.93	1.60 ± 0.92	0.019
**TC (mmol/L)**	4.22 ± 0.95	4.14 ± 0.96	4.35 ± 0.91	0.009
**HDL-C (mmol/L)**	1.32 ± 0.29	1.35 ± 0.30	1.26 ± 0.27	0.008
**LDL-C (mmol/L)**	2.59 ± 0.63	2.52 ± 0.60	2.69 ± 0.66	0.002
**FBG (mmol/L)**	5.04 ± 0.37	5.02 ± 0.39	5.08 ± 0.35	0.166
**FINS (pmol/L)**	85.7 (49.51, 123.21)	55.01 (34.24, 77.18)	143.20 (114.11, 185.40)	<0.001
**HOMA-β(%)**	153.31 (86.73, 257.82)	98.00 (60.39, 144.38)	281.60 (200.32, 357.72)	<0.001
**HOMA-IR**	2.71 (1.52, 4.04)	1.75 (1.10, 2.49)	4.56 (3.76, 5.86)	<0.001

BMI, body mass index; SUA, serum uric acid; TG, triglyceride, TC, total cholesterol; HDL-C, high density lipoprotein-cholesterol; LDL-C, low density lipoprotein-cholesterol; FBG, fasting blood glucose; FINS, fasting insulin; HOMA-**β**, homeostasis model assessment-**β**; HOMA-IR, homeostasis model assessment-insulin resistance.

### Correlation Analysis of BMI and SUA With HOMA-IR

The correlation analysis showed that HOMA-IR was closely associated with BMI, SUA, TG, TC, HDL-C, LDL-C and FINS (all p < 0.05, **Table 2**). To further explore the correlations between HOMA-IR and BMI, HOMA-IR and SUA, the results of the multiple linear regression analysis are presented in **Table 3**. The results indicated that both BMI and SUA were risk factors for HOMA-IR with the crude model and when adjusted for age and sex (all p < 0.05, **Table 3**). However, after adjusting for age, sex, TG, TC, HDL-C, LDL-C, and BMI (p < 0.05, **Table 3**), there was no correlation between SUA and HOMA-IR (p > 0.05, **Table 3**).

**Table 2:** Univariate analysis examining the association of BMI and SUA with HOMA-IR.

	HOMA-IR		SUA	
	r	*P*	r	*P*
**Sex**	0.001	0.979	-0.130	0.012
**Age**	0.310	<0.001	0.497	<0.001
**BMI**	0.338	<0.001	0.461	<0.001
**SUA**	0.255	<0.001	–	–
**TG**	0.243	<0.001	0.108	0.037
**TC**	0.124	0.017	0.156	0.003
**HDL-C**	-0.119	0.022	-0.201	<0.001
**LDL-C**	0.146	0.005	0.004	0.941
**FBG**	0.097	0.063	0.016	0.756
**FINS**	0.993	<0.001	0.261	<0.001
**HOMA-β**	0.834	<0.001	0.259	<0.001
**HOMA-IR**	–	–	0.255	<0.001

BMI, body mass index; SUA, serum uric acid; TG, triglyceride, TC, total cholesterol; HDL-C, high density lipoprotein-cholesterol; LDL-C, low density lipoprotein-cholesterol; FBG, fasting blood glucose; FINS, fasting insulin; HOMA-β, homeostasis model assessment-β; HOMA-IR, homeostasis model assessment-insulin resistance.

**Table 3:** Multiple linear regression examining the association of BMI and SUA with HOMA-IR.

Dependent variables	BMI			SUA	
	Beta	*P*		Beta	*P*
**Model 1**					
HOMA-IR	0.338	<0.001		0.255	<0.001
**Model 2**					
HOMA-IR	0.243	<0.001		0.138	0.016
**Model 3**					
HOMA-IR	0.211	0.001		0.074	0.188

Model 1: crude model;

Model 2: adjusted for age and sex;

Model 3: adjusted for age, sex, TG, TC, HDL-C, and LDL-C.

BMI, body mass index; SUA, uric acid; HOMA-IR, homeostasis model assessment-insulin resistance.

### Correlation Analysis of BMI With SUA

The correlation analysis showed that BMI, TG, TC, HDL-C and FINS were closely associated with SUA (all p < 0.05, **Table 2**). Further multiple linear regression analysis found that BMI was a risk factor for SUA with the crude model adjusted for age and sex and for age, sex, TG, TC, HDL-C, LDL-C, FBG and FINS (all p < 0.05, **Table 4**).

**Table 4:** Multiple linear regression examining the association of BMI with SUA.

Dependent variables	Beta	t	*P*	95% CI
**Model 1**				
SUA	0.461	9.931	<0.001	8.293 to 12.384
**Model 2**				
SUA	0.246	4.410	<0.001	3.057 to 7.977
**Model 3**				
SUA	0.206	3.574	<0.001	2.082 to 7.175

Model 1: crude model;

Model 2: adjusted for age and sex;

Model 3: adjusted for age, sex, TG, TC, HDL-C, LDL-C, FBG, and FINS.

BMI, body mass index; SUA, serum uric acid; TG, triglyceride, TC, total cholesterol; HDL-C, high density lipoprotein-cholesterol; LDL-C, low density lipoprotein-cholesterol; FBG, fasting blood glucose; FINS, fasting insulin; CI, confidence interval.

### Direct and Indirect Effects of BMI on Markers of Glucose Metabolism With SUA As a Mediator

As displayed in **Table 5**, the mediation analysis based on a causal pathway revealed the mediating role of SUA on the relationship between BMI and glucose metabolism. BMI was directly associated with FINS and HOMA-IR [both p (c’)< 0.05, **Table 5**]. In addition, the potential causal effect of BMI on FINS and HOMA-IR mediated by SUA was also presented [All p (a) and p (b)< 0.05, **Table 5**], which implied that SUA could have a mediating impact on the link between BMI and glucose metabolism. However, BMI was not directly associated with FBG and not indirectly associated with FBG with SUA as a mediator (all p > 0.05, **Table 5**).

**Table 5:** Direct and indirect effects of BMI on markers of glucose metabolism with SUA as a mediator.

Outcomes	Direct effect (SUA unadjusted)		Indirect effect (SUA adjusted)			
	Estimate^1^ (BMI to glucose metabolism)	*P*(c')	Estimate^2^ (BMI to SUA)	*P*(a)	Estimate^3^ (SUA to glucose metabolism)	*P*(b)
**FBG**	-0.004	0.310	0.460	<0.001	0.002	0.457
**FINS**	4.476	<0.001	0.460	<0.001	0.086	0.021
**HOMA-IR**	0.138	<0.001	0.460	<0.001	0.002	0.022

^1^Estimate represents the direct effect of BMI on markers of glucose metabolism.

^2^Estimate represents the indirect effect of BMI on SUA.

^3^Estimate represents the indirect effect of SUA on markers of glucose metabolism.

BMI, body mass index; SUA, serum uric acid; FBG, fasting blood glucose; FINS, fasting insulin; HOMA-IR, homeostasis model assessment-insulin resistance.

## Discussion

This study has demonstrated several unexpected findings with important clinical implications. The results showed that obesity had direct impacts on regulating FINS and HOMA-IR, and the results of mediating effect analysis also highlighted that the link between obesity and IR was partly mediated by SUA in obese children and adolescents. However, the present study did not find that obesity directly or indirectly affects FBG.

Obesity is an ongoing global epidemic that severely affects adults and children. Many risk factors for IR have been identified in obese children and adolescents, and one of them is serum uric acid. However, it remains controversial whether SUA plays a pathogenic role in IR. In our study, it was found that SUA was a risk factor for IR and exerted a mediating role in the process of obesity-induced IR, which implied that SUA may not only directly affect IR but also be the intermediate link leading to IR. That is, a BMI → SUA → IR pathway is present in obese individuals.

It is known that obesity is strongly associated with IR in obese children and adolescents (10, 20), which was in line with our study. However, the mechanisms of obesity leading to IR are not fully understood to date. There has been growing evidence suggesting that IR is induced through chronic low-grade inflammation, which is promoted by adipocytokines (21–23). A previous study suggested that leptin and adiponectin could modulate the relationship between obesity and IR (24). Other studies also showed a positive association between leptin and IR after controlling for potential confounding factors in children and adolescents (22, 25). Thus, these studies could indicate that adipocytokines are the direct pathway of obesity-insulin resistance.

Furthermore, cross-sectional studies showed that an excessive increase in BMI and waist circumference was associated with significant SUA elevation and hyperuricemia (26–28). It has been demonstrated that mature adipocytes and adipose tissue produce and secrete uric acid (29). Thus, obesity could increase the mRNA expression and activity of xanthine oxidoreductase from adipose tissue (29, 30).

Viewed separately, a close relationship between SUA and IR has long been appreciated, which may be another mechanism to explain IR in obese people. Emerging evidence has shown that hyperuricemia is linked with IR and the subsequent promotion and development of T2DM (9, 31, 32). Similar to these studies, we found that the SUA level was higher in the high HOMA-IR group, and SUA was also an independent risk factor for HOMA-IR. Furthermore, another major finding of the present study was that advanced causal mediating analysis supported that IR was partly mediated by SUA, which implies that an obesity → SUA → IR indirect pathway exists in obese children and adolescents.

Regarding the mechanism involved, the causal relationship between hyperuricemia and IR has not been completely illuminated and is still under investigation. In an animal study with eight-week-old male C57BL/6J mice, increased SUA levels might inhibit IRS1 and Akt insulin signaling and induce IR, which indicates a key role of the reactive oxygen species pathway in high SUA-induced IR (29). There are also reports that SUA-induced IR is caused by an increase in tissue NADPH oxidase or hs-CRP levels (33–35).

In the present study, there was no direct or indirect correlation between obesity and FBG. This result is not surprising, as IR precedes the development of diabetes. This hints that obese children oversecrete insulin in response to increasing insulin resistance to maintain the FBG in the normal range. However, this condition is strongly associated with alterations that represent an increased risk for the development of metabolic disorders and the occurrence of diabetes in adulthood (3, 9).

Nevertheless, there are several limitations in this study that deserve comment. First, because of the retrospective study design, other potential mediators could not be fully taken into account. Second, as obesity in our study was evaluated by BMI, which cannot differentiate excess body weight from increased fat mass or fat-free mass, further studies will be conducted to investigate the effects of different body compositions on hyperuricemia and IR.

In conclusion, SUA plays a partial mediating role in IR induced by obesity in obese children and adolescents. SUA should be investigated in obesity, and the results of the present study indicate this avenue is worth pursuing. In addition, the long-term direct and indirect effects of obesity and SUA on FBG need to be investigated in the future.

## Data Availability Statement

The raw data supporting the conclusions of this article will be made available by the authors, without undue reservation.

## Ethics Statement

This study was reviewed and approved by the Ethic Committee of Xinhua Hospital, School of Medicine, Shanghai Jiao Tong University (No. XHEC-D-2021-113). Written informed consent to participate in this study was provided by the participants’ legal guardian/next of kin.

## Authors Contributions

YN, QT, XuaZ, WC, and YF conceptualized and designed the study, drafted the initial manuscript, and reviewed and revised the manuscript. XueZ, XM, and JS designed the data collection instruments, collected data, carried out the initial analyses, and reviewed and revised the manuscript. All authors contributed to the article and approved the submitted version.

## Conflict of Interest

The authors declare that the research was conducted in the absence of any commercial or financial relationships that could be construed as a potential conflict of interest.

## Publisher’s Note

All claims expressed in this article are solely those of the authors and do not necessarily represent those of their affiliated organizations, or those of the publisher, the editors and the reviewers. Any product that may be evaluated in this article, or claim that may be made by its manufacturer, is not guaranteed or endorsed by the publisher.
